# Identification of TYR, TYRP1, DCT and LARP7 as related biomarkers and immune infiltration characteristics of vitiligo via comprehensive strategies

**DOI:** 10.1080/21655979.2021.1933743

**Published:** 2021-06-09

**Authors:** Jiayu Zhang, Rongguo Yu, Xiaoyu Guo, Yuanxia Zou, Sixuan Chen, Kai Zhou, Yi Chen, YongRong Li, Su Gao, Yifei Wu

**Affiliations:** a Department of Dermatology, the First People’s Hospital of Yunnan Province, The Affiliated Hospital of Kunming University of Science and Technology, Kunming, Yunnan, China; b Department of Dermatology, The First Clinical Medical College of Yunnan University of Traditional Chinese Medicine, Kunming, Yunnan, China; c Department of Orthopedics, Fuzhou the Second Hospital Affiliated to Xiamen University, Fujian, China; d Department of Neurosurgery/Neuro-oncology, Sun Yat-sen University Cancer Center, State Key Laboratory of Oncology in South China, Collaborative Innovation Center for Cancer Medicine, Guangzhou, China; e Department of Newborn Medicine, Hospital (T.C.M) Affiliated to Southwest Medical University, Luzhou, Sichuan, China

**Keywords:** Vitiligo, machine learning algorithm, immune cells, biomarkers, cibersort

## Abstract

This study aims to explore biomarkers associated with vitiligo and analyze the pathological role of immune cell infiltration in the disease. We used the robust rank aggregation (RRA) method to integrate three vitiligo data sets downloaded from gene expression omnibus database, identify the differentially expressed genes (DEGs) and analyze the functional correlation. Then, the comprehensive strategy of combined weighted gene coexpression network analysis (WGCNA) and logical regression of the selection operator (LASSO), support vector machine recursive feature elimination (SVM-RFE), and random forest (RF) machine learning algorithm are employed to screen and biomarkers associated with vitiligo. Finally, the immune cell infiltration of vitiligo was evaluated by CIBERSORT, and the correlation between biomarkers and infiltrating immune cells was analyzed. Herein, we identified 131 robust DEGs, and enrichment analysis results showed that robust DEGs and melanogenesis were closely associated with vitiligo development and progression. TYR, TYRP1, DCT and LARP7 were identified as vitiligo-related biomarkers. Immune infiltration analysis demonstrated that CD4 T Cell, CD8 T Cell, Tregs, NK cells, dendritic cells, and macrophages were involved in vitiligo’s pathogenesis. In summary, we adopted a comprehensive strategy to screen biomarkers related to vitiligo and explore the critical role of immune cell infiltration in vitiligo.

**Abbreviations**: TYR, Tyrosinase; TYRP1, Tyrosinase-related protein-1; DCT, dopachrome tautomerase; LARP7, La ribonucleoprotein domain family, member-7; RRA, robust rank aggregation; DEGs, differentially expressed genes; WGCNA, weighted gene coexpression network analysis; LASSO, logical regression of the selection operator; SVM-RFE, support vector machine recursive feature elimination; RF, random forest; GWAS, Genome-wide association study; FasL, Fas-Fas ligand; Tregs, T-regulatory cells; NK, natural killer; GEPCs, gene expression profiling chips; GO, gene ontology; GSEA, gene set enrichment analysis; FDR, false discovery rate; AUC, area under the curve; ROC, receiver-operating characteristic; BP, biological process; CC, cellular component; MF, molecular function.

## GraphicalAbstract

**Figure:**
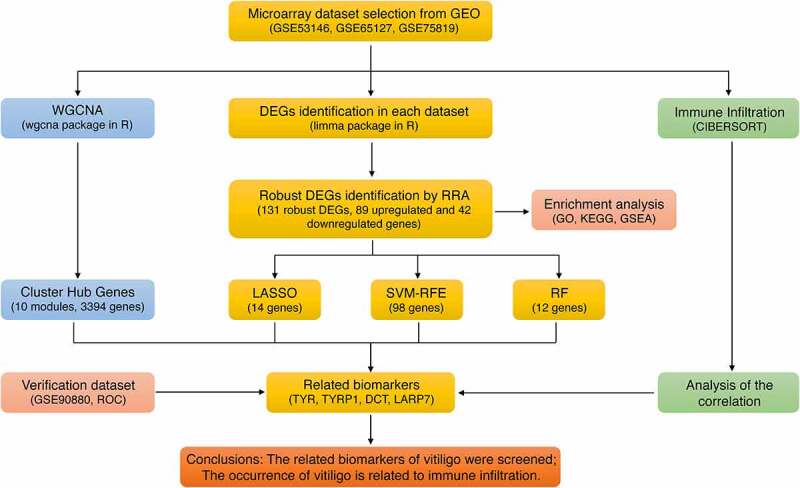

## Introduction

Vitiligo is an autoimmune dermatological disease characterized by the destruction of melanocytes and chronic depigmentation, resulting in gradual white patches in the skin. Globally, the prevalence rates ranged from 0.5% to 2% approximately [1,2]. The etiology of vitiligo is very complex, involving genetic predisposition, oxidative stress, environmental triggers, metabolic abnormalities, impaired renewal, and altered inflammatory and immune responses. Genome-wide association study (GWAS) has identified multiple genetic loci, which increased vitiligo risks, including gene polymorphism, immunity, antigen presentation, melanocytes, and peptidases [3]. Systematic reviews have shown that histopathological and serological diagnoses revealed some biomarkers associated with disease activity in vitiligo. These biomarkers include cytokines (IL-1β, IL-17, IFN-γ, TGF-β), autoantibodies, oxidative stress markers, immune cells, and antibodies (RCLs), soluble CDs (sCD25, sCD27), and chemokines (CXCL9, CXCL10) [4]. However, the relationship between these biomarkers and the pathogenesis of vitiligo is not fully clear.

Vitiligo is associated with polymorphisms in genes involved in the immune response and in melanogenesis [5]. Recent immunological studies have extensively proved that immune cell infiltration plays a vital role in the occurrence and development of vitiligo. For instance, melanocyte-specific CD8 + T cells are enriched in the peri-lesional skin of vitiligo in vitro studies [6]. CD8^+^ cytotoxic T cells can cause melanocyte injury [7], and the ratio of CD8 + T cells increases in lesional skin and peripheral blood of vitiligo patients [8,9]. CD8 + T cells can directly induce cytolysis of target cells by releasing soluble cytotoxic molecules and Fas-Fas ligand (FasL) interaction, inducing skin depigmentation cytotoxic T-cell response [10]. In addition to CD8 + T cells, many kinds of immune cell infiltration are found, including CD4+ Tcells, T-regulatory cells (Tregs), natural killer (NK) cells, dendritic cells, and skin resident T cells in the blood and skin lesions of vitiligo patients [11,12]. The degree of infiltration of these immune cells is highly correlated to activity and severity of vitiligo. However, the immunopathological mechanism of vitiligo remains imprecise. Therefore, evaluating immune cell infiltration degree in vitiligo and exploring changes in relative abundance of immune cell types of potential associated markers to further elucidate the molecular mechanism underlying vitiligo and develop new immunotherapy targets is highly significant.

As a classical bulk RNA deconvolution algorithm analysis tool, CIBERSORT is usually employed to evaluate immune cell infiltration degree in tissue samples. CIBERSORT has been used in skin diseases to analyze immune cell infiltration characteristics in malignant melanomas [13], acne [14], atopic dermatitis, and psoriasis [15]. However, we have not found any other studies that utilize CIBERSORT algorithm to analyze immune cell infiltration abundance in vitiligo and evaluate its value. In this study, we used CIBERSORT for the first time to analyze the difference of immune infiltration between lesional and normal tissues in 22 immune cell subsets.

Thus, this study aimed to further screen and determine biomarkers related to vitiligo using machine learning integrated strategies and weighted gene coexpression network analysis. In addition, we also used the CIBERSORT algorithm to study the changes in differences between biomarkers and infiltrated immune cells to understand better the molecular immune mechanisms involved in vitiligo. We hope to provide directions for further research via a variety of algorithm strategies to identify associated markers of skin diseases.

## Materials and methods

### Data collection and data processing

We selected three vitiligo gene expression microarray (gene expression profiling chips, GEPCs) from the GEO database [16], including GSE53,146, GSE65127, and GSE75819. The RMA algorithm was applied to background correction and data normalization [17]. The samples’ inclusion criteria were as follows: lesional skin and normal skin of patients with vitiligo, excluding non-lesional and peri-lesional skin tissue. GSE53146 contains 5 lesional skin and 5 normal skin, GSE65127 contains 10 lesional skin and normal skin, and GSE75819 comprises 15 lesional skin and normal skin. These lesional skin were mainly from the upper back, abdomen, forearm, lower leg, and thigh. Then, DEGs identified each dataset through ‘*limma*’ package [18], and the volcano plot of DEGs was drawn to show their differential expression. DEGs with *p* < 0.05 and |log_2_FC| > 1 were considered statistically significant.

### Robust rank aggregation analysis

The RRA method was utilized to integrate three datasets, identify robust DEGs [19], and minimize the deviation and error between multiple datasets. The upregulated and downregulated genes were sequenced in each dataset. RRA package is executed to obtain robust DEGs based on the ranked genes in the three datasets. FC > 1 and *P*-value < 0.05 were considered to be truncation criteria for significant robust DEGs. We rectified the expression matrices of GSE53146, GSE65127, and GSE75819 datasets to standardize the data and merge them into an independent dataset after quality control RRA analysis.

### Functional correlation analysis

The ‘*clusterProfiler*’ package was used for gene ontology (GO) and KEGG enrichment analysis to identify the function of robust DEGs [20]. The gene set enrichment analysis (GSEA) of gene expression matrix was conducted by ‘*clusterProfiler*’ package, and ‘c2.cp.kegg.v7.2.symbols.gmt’ was selected as the reference gene set [21]. A false discovery rate (FDR) < 0.25 and *p* < 0.05 were considered significant enrichment.

### Screening characteristic related biomarkers via the comprehensive strategy

We combined WGCNA, logical regression of the selection operator (LASSO) [22], support vector machine recursive feature elimination (SVM-RFE) [23], and random forest (RF) [24] to analyze vitiligo-related biomarkers. WGCNA was employed to find DEG modules with high correlations to vitiligo, and RF was utilized for supervised machine learning. The least absolute shrinkage and LASSO were combined with the feature selection of SVM-RFE to find vitiligo-related biomarkers. WGCNA was used to identify highly synergistic gene sets and identify biomarkers based on interconnectedness of gene sets and association between gene sets and phenotypes [25]. SVM-RFE was a machine learning method based on a support vector machine used to find the best variables by deleting feature vectors generated by SVM and further identifying these biomarkers’ associated value in vitiligo through ‘*e1071*’ package [26]. RF was a widely used machine learning algorithm based on decision tree theory. The RF algorithm was a machine learning algorithm based on decision tree theory classified according to its ability to deal with high-dimensional data and select the most informative gene clusters. The *pROC* R package [27] was employed to compute the area under the curve (AUC) of a receiver-operating characteristic (ROC) curve to evaluate the joint associated efficiency of biomarkers. *P* < 0.05 was considered to be statistically significant.

### Evaluation and correlation analysis of immune infiltrating cells

The CIBERSORT algorithm was utilized to filter 22 kinds of immune cell matrix [28]. According to *p* < 0.05, the immune cell infiltration matrix was obtained. The ‘*ggplot2*’ package was employed for PCA cluster analysis of immune cell infiltration matrix [29]. The ‘*corrplot*’ package was deployed to draw the correlation heatmap to visualize the correlation of 22 kinds of immune cell infiltration [30]. The ‘*ggstatsplot*’ (https://github.com/IndrajeetPatil/ggstatsplot) and ‘*ggplot2*’ packages were used to analyze the Spearman correlation between characteristic biomarkers and immune infiltrating cells and visualize the results.

## Results

Although many biomarkers of vitiligo have been identified in previous studies, the relationship between the immune infiltration characteristics and these biomarkers of vitiligo remained unclear. Thus, this study aimed to screen potential biomarkers related to vitiligo by using multiple machine learning integrated strategies (LASSO, SVM-RFE, RF) and WGCNA analysis. To understand better the molecular immune mechanisms involved in vitiligo, we also used the CIBERSORT algorithm to study the changes in differences between biomarkers and infiltrated immune cells. Ultimately, TYR, TYRP1, DCT, and LARP7 have been screened as candidate biomarkers associated with vitiligo. In addition, the immune infiltration characteristics of these biomarkers were analyzed.

### Screening of DEGs in different datasets

**Figure 1.**
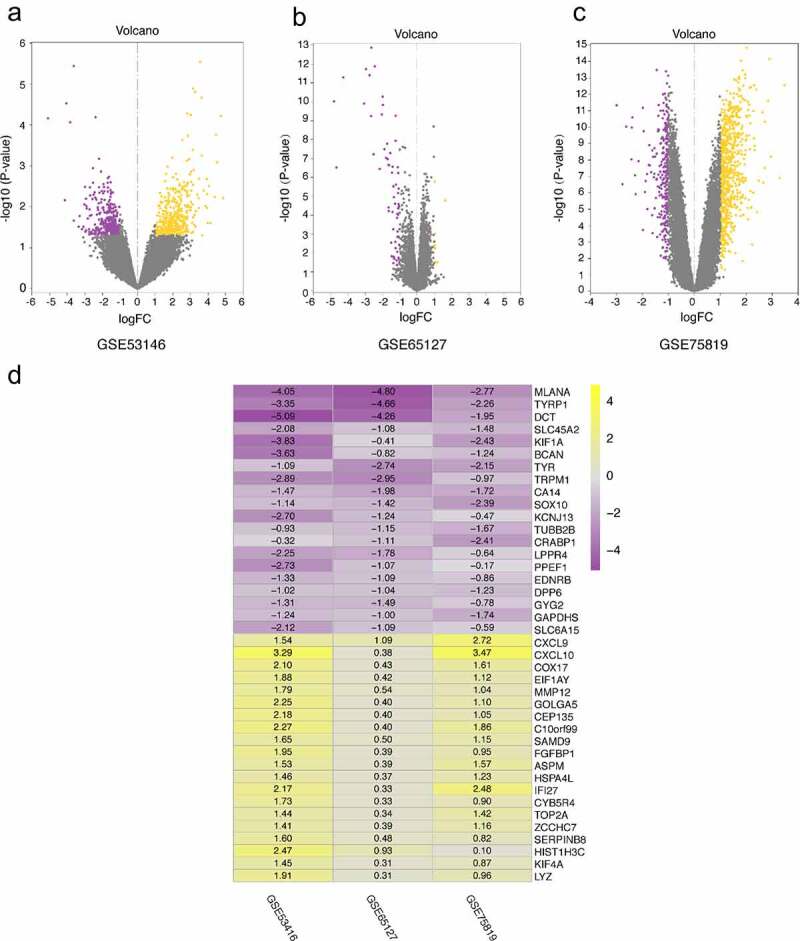
Volcano plots of DEGs distribution in GSE53146 (a), GSE65127 (b) and GSE75819 (c). the yellow and purple dots represent upregulated and downregulated genes, respectively. (d) the heatmap of top 20 upregulated and downregulated robust DEGs identified by RRA method. yellow represents a high expression of robust DEGs, while purple represents a low expression of robust DEGs

The DEGs of GSE53146, GSE65127 and GSE75819 were identified by *limma* package. According to cutoff criteria of |log_2_FC| > 1 and *P* < 0.05, there were 847 DEG in GSE 53146, including 412 upregulated and 435 downregulated genes. A total of 73 DEGs were screened from GSE65127 data set, including 9 upregulated and 64 downregulated genes. A total of 1064 DEGs were selected in GSE75819, including 777 upregulated and 287 downregulated genes. The volcano plot was used to show DEGs in different data sets (Figure 1a-c).

### Identification of robust DEGs by robust rank aggregation

A total of 131 robust DEGs were identified by RRA method, including 89 upregulated and 42 downregulated genes. We allocated the top 20 upregulated and downregulated robust DEGs in visual heatmap according to *P*-value < 0.05 (Figure 1d).

### Functional enrichment analyses of robust DEGs

**Figure 2.**
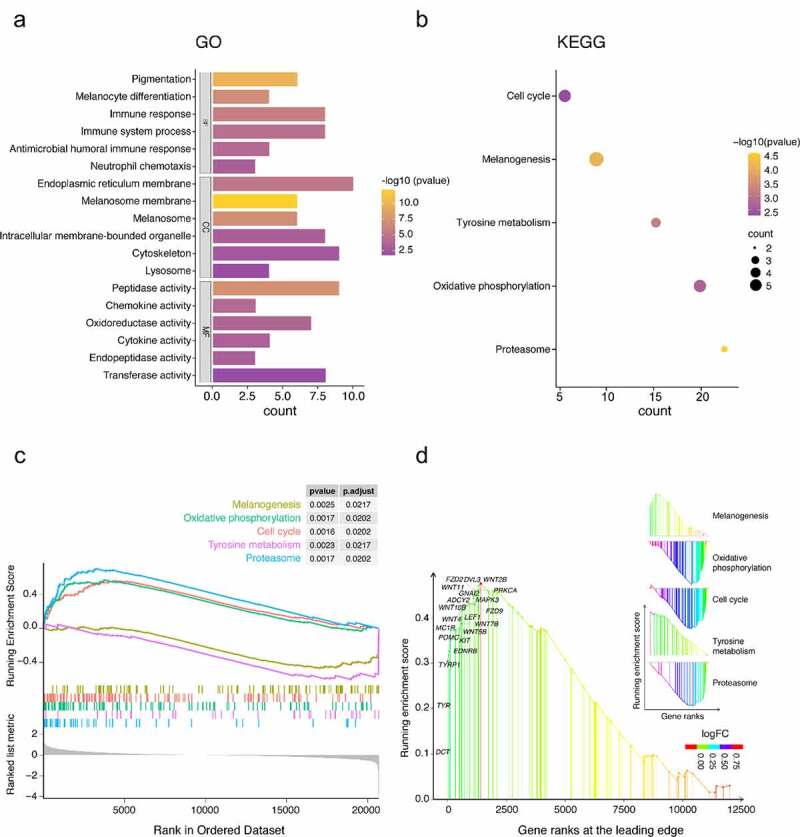
Functional enrichment analysis of robust DEGs. (a) GO enrichment analysis and its BP, CC, and MF three parts. (b) KEGG enrichment analysis. (c) GSEA showed that the top 5 signal pathways were most related to vitiligo pathology. (d) rank-based enrichment analysis visualized five signal pathways and showed melanogenesis signal pathways and gene ranks at the leading edge

The GO analysis shows the top five most relevant terms. For biological process (BP), GO analysis showed that robust DEGs were mainly concentrated in pigmentation, melanocyte differentiation, immune response, immune system process, neutrophil chemotaxis, antimicrobial humoral immune response mediated by antimicrobial peptide, and killing of cells of other organism signal pathways. The cellular component (CC) part, melanosome, is mainly enriched in melanosome membrane, endoplasmic reticulum membrane, cytoskeleton, intracellular membrane-bounded organelle, and lysosome. The significantly enriched term in molecular function (MF) group was peptidase, transferase, oxidoreductase, cytokine, chemokine and endopeptidase activities (Figure 2a). The KEGG and GSEA analysis results showed that melanogenesis, oxidative phosphorylation, cell cycle, tyrosine metabolism, and proteasome signal pathways were highly related to vitiligo pathology (Figure 2b-c). These signaling pathways and gene ranks at the leading edge were visualized by rank-based enrichment analysis and ‘*ggplot2*’ package (Figure 2d).

### Screening characteristic related biomarkers via the comprehensive strategy

**Figure 3.**
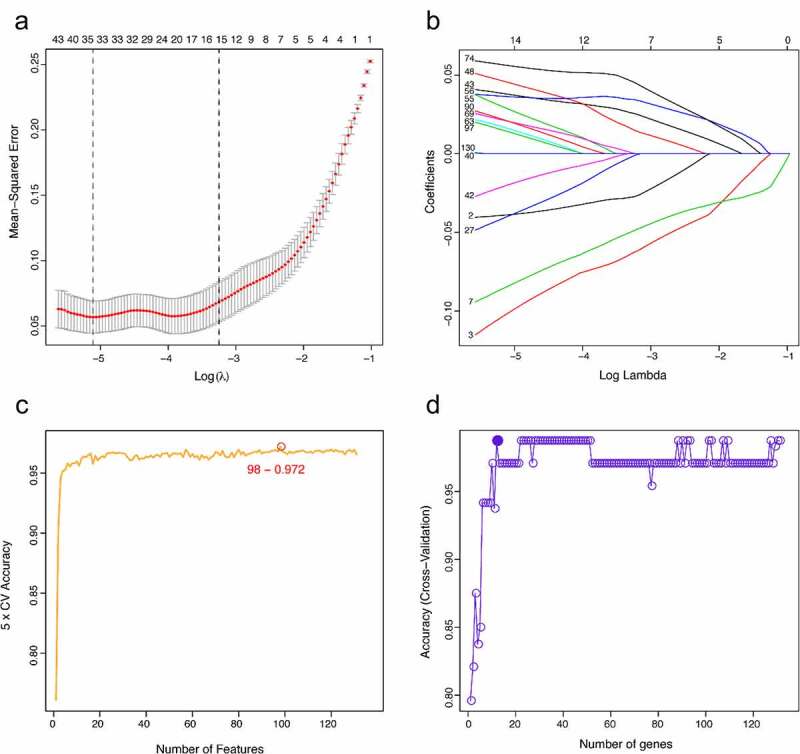
Screening characteristic related biomarkers via comprehensive strategy. (a) the least absolute shrinkage and selection operator (LASSO) logistic regression algorithm is used to retain the most predictive features. (b) different colors represent different genes. based on support vector machine recursive feature elimination (SVM-RFE) algorithm (c) and random forest (RF) algorithm (d) to screen biomarkers

**Figure 4.**
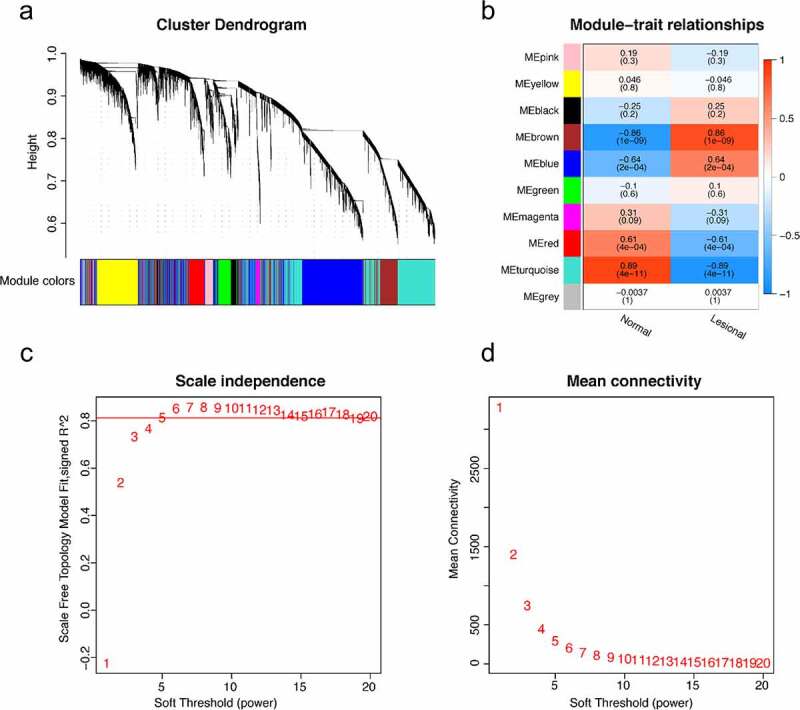
(a) the cluster dendrogram of genes in independent data sets. the branching of clustering dendrograms of the most closely connected genes produced 10 gene coexpression modules. (b) relationships of consensus modules with samples. it contains a set of highly linked genes. each specified color represents a specific gene module. (c) analysis of the scale-free fit index for various soft-thresholding powers (beta). the red line represents merging threshold. (d) the mean connectivity of various soft threshold power was analyzed

**Figure 5.**
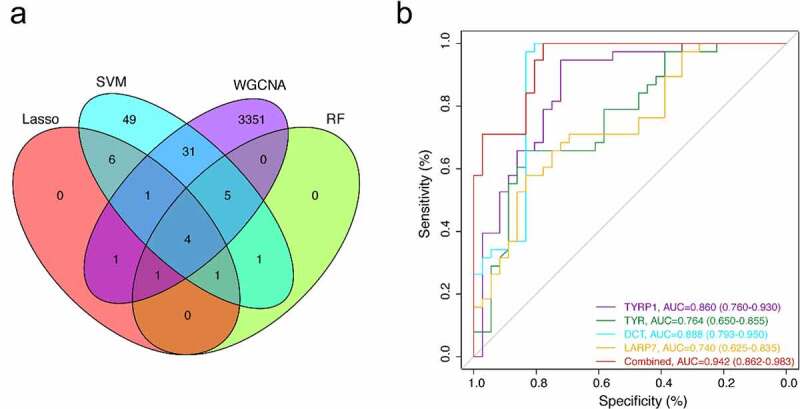
(a) the venn diagram showed the intersection of biomarkers obtained by four algorithms. (b) four associated markers were fitted into one variable, and ROC curve was used to verify the associated efficiency

The LASSO logistic regression algorithm was used to identify 14 genes from robust DEGs as potential vitiligo-related biomarkers (Figure 3a-b). Ninety-eight genes were identified as potential biomarkers from robust DEGs by SVM-RFE algorithm (Figure 3c). Twelve genes were identified from robust DEGs using RF algorithm (Figure 3d). To further improve screening characteristic biomarkers’ accuracy, we used WGCNA to analyze independent data sets merged by quality control to identify the modules containing highly correlated genes. The soft‐threshold power 5 was chosen to ensure that criterion of approximate scale-free topology (Figure 4a-b). We set MEDissThres as 0.25 to merge similar modules and generated 10 modules (Figure 4c-d). The hub genes were obtained in turquoise module, which is highly related to developing the disease. Finally, the gene markers obtained by the four algorithms were overlapped, and the related biomarkers, including TYR, TYRP1, DCT, and LARP7, were obtained (Figure 5a). We used GSE90880 dataset as the verification set to validate the efficacy of four related biomarkers. The four biomarkers’ associated efficiency in the verification set reached a significant level (AUC = 0.942), indicating a high associated value (Figure 5b).

### Analysis of Immune Infiltrating Cells

**Figure 6.**
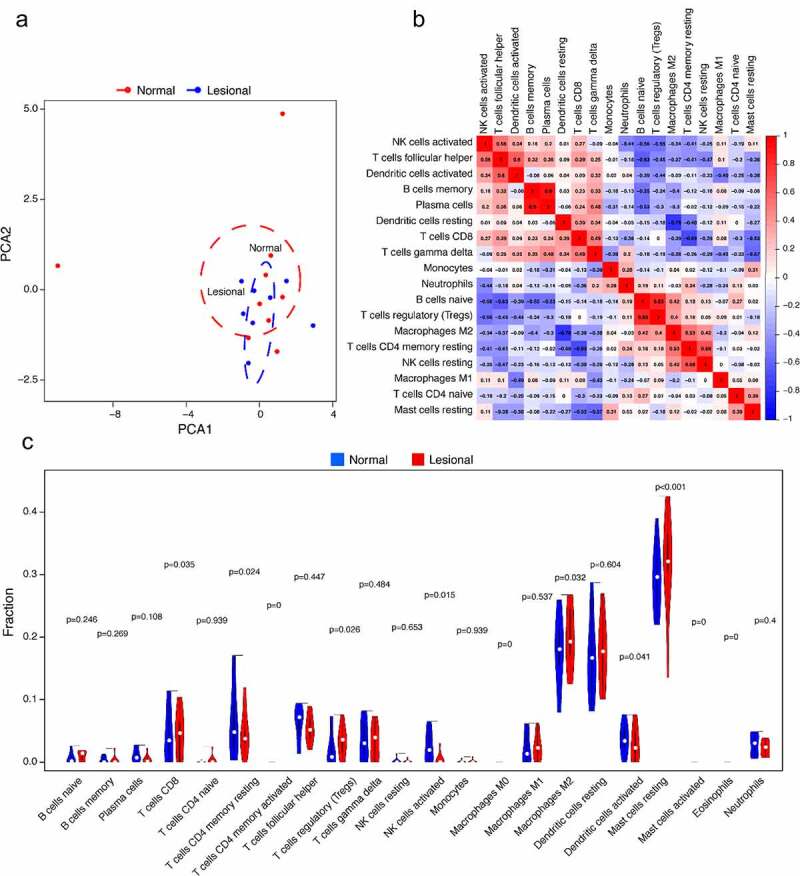
Immune cells infiltration analysis. (a) PCA results of immune infiltration between lesional and normal samples. (b) The correlation heatmap showed 22 kinds of immune cell infiltration, and 4 kinds of immune cells with no difference were removed. The size of color square represents correlation intensity, red represents the positive correlation, and blue represents the negative correlation. (c) The violin plot showed the difference of 22 kinds of immune cell infiltration between two groups. The red markers represent immune cells with significant differences in infiltration

The PCA cluster analysis results displayed differences in some immune infiltration between normal and lesional samples (Figure 6a). The CIBERSORT algorithm showcased the infiltration of 22 kinds of immune cells that there were no significant differences in the infiltration of immune cells between T cells CD4 memory activated, macrophages M0, mast cells activated, and eosinophils. The correlation heatmap exhibited a positive correlation between plasma cells and B cells memory in the immune infiltrating cells of vitiligo. A positive correlation was found between T cells regulatory (Tregs) and B cells naive, and a positive correlation was present between NK cells resting and T cells CD4 memory resting. While macrophages M2 and dendritic cells resting have a negative correlation, T cells CD4 memory resting and T cells CD8 also negatively correlate (Figure 6b). The violin plot also manifested that the immune infiltration of Tregs and mast cells resting was more, while that of plasma cells and NK cells activated was less (Figure 6c).

### Analysis of the correlation between related biomarkers and immune infiltrating cells

**Figure 7.**
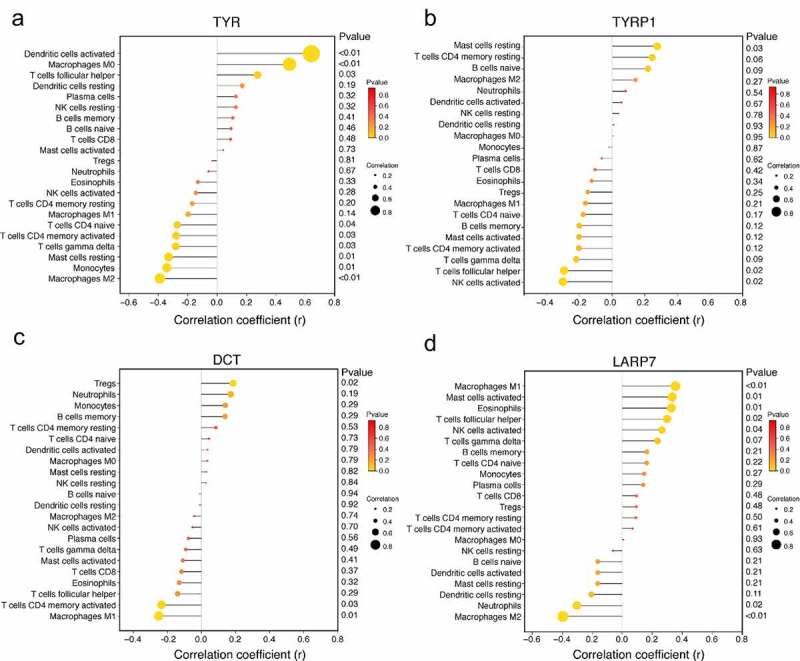
Analysis of the correlation between biomarkers and infiltrating immune cells. (a) Correlation between TYR and infiltrating immune cells. (b) Correlation between TYRP1 and infiltrating immune cells. (c) Correlation between DCT and infiltrating immune cells. (d) Correlation between LARP7 and infiltrating immune cells. The dot size represents correlation intensity between genes and immune cells. The lower the *p*-value, the more yellow the color, and the higher the *p*-value, the redder the color

Correlation analysis showed that TYR was positively correlated with dendritic cells activated (r = 0.644, *p* < 0.01), macrophages M0 (r = 0.495, *p* < 0.01) and T cells follicular helper (r = 0.278, *p* = 0.03) . TYR was negatively correlated with mast cells resting (r = −0.328, *p* = 0.01), monocytes (r = −0.34, *p* = 0.01) and macrophages M2 (r = −0.387, *p* < 0.01) (Figure 7a). TYRP1 was positively correlated with mast cells resting (r = 0.276, *p* = 0.03), and negatively correlated with T cells follicular helper (r = −0.296, *p* = 0.02) and NK cells activated (r = −0.303, *p* = 0.02) (Figure 7b). DCT was positively correlated with Tregs (r = 0.316, *p* = 0.02), and negatively correlated with T cells CD4 memory activated (r = −0.269, *p* = 0.03) and macrophages M1 (r = −0.367, *p* = 0.01) (Figure 7c). LARP7 was positively correlated with macrophages M1 (r = 0.354, *p* < 0.01), mast cells activated (r = 0.332, *p* = 0.01) and eosinophils (r = 0.326, *p* = 0.01). LARP7 was negatively correlated with neutrophils (r = −0.304, *p* = 0.02) and macrophages M2 (r = −0.398, *p* < 0.01) (Figure 7d).

## Discussion

Vitiligo is a common immune-mediated depigmented disease that is easy to diagnose but with a long treatment cycle. Although vitiligo has little impact on physical health, it seriously affects patient beauty and causes a substantial psychological burden. To further understand the molecular mechanisms of the occurrence and development of vitiligo, we identified 131 robust DEGs using RRA method, including 89 upregulated genes and 42 downregulated genes. GO enrichment analysis revealed that robust DEGs were mainly related to pigmentation, melanocyte differentiation, immune response, and other immune cell signal pathways. The enrichment pathways of KEGG and GSEA are mainly correlated with melanogenesis, oxidative phosphorylation, cell cycle, tyrosine metabolism, and proteasome signal pathways. Furthermore, rank-based enrichment analysis showed significantly more modulated genes in GSEA analysis. Previous studies have shown that these potential signaling pathways are not only related to melanin production and metabolism, but also involved in the development of vitiligo [31,32].

As a system biology method, constructing WGCNA and identifying gene clusters or modules help to explore the characteristic relationship between disease and gene clusters. Meanwhile, we integrated the machine learning algorithm to improve the accuracy of screening biomarkers. LASSO logistic regression determines variables by exploring λ. SVM-RFE selects variables and explains direction and strength of correlation between predictors and outcomes by recursive feature elimination of non-linear kernels [33]. RF can deal with unbalanced and missing values in data. These three machine learning algorithms are mainly used to screen feature variables and establish the best classification model. Herein, TYR, TYRP1, DCT, and LARP7 were chosen as biomarkers to identify vitiligo by combining machine learning algorithm and WGCNA.

Tyrosinase (TYR) is a critical enzyme in melanin metabolism, with abnormal expression closely related to vitiligo, melanoma, and Parkinson’s disease. Tyrosinase-related protein-1 (TYRP1) is melanocyte-specific enzymes involved in melanin biosynthesis. Melanocyte-specific markers involved in melanin synthesis are mainly responsible for tyrosine-to-melanin conversion, including tyrosinase-related protein-(TRP-) 1, TRP-2, and tyrosinase. The destruction of melanocytes, dysfunction, and obstruction of synthesis pathways are the leading causes of vitiligo. In human melanocytes, the cyclic adenosine monophosphate/protein kinase A (cAMP/PKA) pathway, as one of the mediators, can promote the signal transfer from melanin system to melanogenesis enzymes dopachrome tautomerase (DCT), TYR, and TYRP1, which is regulated by both Wnt and MAPK signal pathways [34]. Recent studies showed that increased cAMP signaling could promote melanoma tumor progression. H_2_O_2_-induced ATP synthase β induces melanogenesis by activating PAH and cAMP/CREB/MITF signaling in melanoma cells [35]. The melanocyte-specific melanocortin-1 receptor (MC1R) binds to α-melanocyte-stimulating hormone (α-MSH) and activates adenylate cyclase, resulting in increased intracellular cAMP levels [36]. These melanin-synthesized genes are regulated by a microphthalmia-associated transcription factor (MITF), modulating melanocytes’ differentiation and leading to an elevated TYR level, TRP1 and TRP2 [37].

The expression of TRP-2, also known as DCT, DCT is regulated by MITF and can be secreted by normal melanocytes and glial cells and presented in MHC class I-presentation on CD8 + T cells [38]. Both DCT and TYR can be expressed in mature melanocytes related to proliferation, migration, and differentiation of melanocyte precursors [39]. Vitiligo is associated with primary allele of SNP in TYR region [40]. TYR, TYRP1, TYRP2, and DCT mRNA levels in vitiligo patients are significantly downregulated and can lead to decreased melanin synthesis [41,42]. Another study on melanocyte autophagy found that the expression of MITF, TYR, TYRP1, and TYRP2 proteins decreased in vitiligo patients [43]. Previous studies have reported that melanocyte signal pathway is one of the core signal pathways in vitiligo, dominating melanocyte production, destruction, and pigment transport as transcription factors of melanocyte signal pathway, TYR, TYRP1, and DCT involved in vitiligo progression.

La ribonucleoprotein domain family, member-7 (LARP7), belongs to LARP RNA binding protein family and is BRCA1 ubiquitinase substrate regulating the metabolism and function of many RNA species and inhibit the occurrence of gastrointestinal tumors [44]. In the zebrafish melanoma model study, it was found that knockout LARP7 could rescue melanocyte gene expression and observe melanocytes in knockout HEXIM1 [45]. Therefore, we speculate that LARP7 is involved in transcriptional regulation and proliferation of melanocytes. However, the current research is still limited, and many clinical studies are required to confirm the associated value of biomarkers.

We used CIBERSORT to further evaluate the immune infiltration of vitiligo to explore the role of immune cell infiltration in vitiligo. CD4 T Cell Subset plays a significant role in coordinating adaptive immune response and participates in pigmentation [46]. CD8 T cells can produce a cytotoxic reaction to melanocytes. CD8 T cells in the skin around the lesions of vitiligo patients can recognize melanocyte antigens and induce autologous melanocyte apoptosis [47]. CD4 and CD8 T cells mainly produce IFN-γ and TNF-α in vitiligo. The activation of CD8 T cells is related to damage of Tregs. Tregs can reduce the proliferation and cytokine production of autoreactive CD8 T cells. Treg cells contain a subset of CD4 + T cells characterized by the expression of FOXP3 transcription factors, which can locally proliferate and dampen skin effector memory T cell responses [10]. The induced expression of CCL22 in the skin increased Tregs infiltration and decreased pigmentation.

The impairment of Tregs’ number and function is closely linked to immune tolerance of vitiligo [11]. NK cells can be activated by local inflammation of vitiligo and drive adaptive immune response by releasing pro-inflammatory cytokines. NK cells have cytotoxicity, which can affect antigen presentation and stimulate the function and maturation of dendritic cells [47]. Macrophages are involved in clearing melanocytes in vitiligo. Macrophage infiltration has been demonstrated in vitiligo lesions, and increased macrophage numbers are also observed in perilesional skin [48]. Besides, our analysis revealed the details of infiltration of 22 kinds of immune cells in vitiligo. However, further experimental data are required to confirm the complex interaction between vitiligo and immune infiltration of biomarkers.

In this study, for the first time, we adopted a comprehensive strategy of WGCNA and machine learning algorithm to screen the biomarkers associated with vitiligo and employed CIBERSORT to analyze the immune cell infiltration of vitiligo. CIBERSORT analysis is based on limited genetic data, and the analysis of immune cell infiltration is still limited. Although some of the previous research results are consistent with our analysis results, the reliability of research results still needs to be verified by further experiments. In addition, the site of the lesional skin has a significant impact on prognosis, such as the fingers and toes, palms and soles, lips, eyelids, nipples and areolas, elbows and knees, and genitals, are considered to be difficult-to-treat areas. An another limitation in this study is the lack of relevant information on the site of the lesional skin. In the subsequent study, we should consider the influence of the lesion anatomical site of the vitiligo on the treatment effect.

## Conclusions

In summary, we found that TYR, TYRP1, DCT, and LARP7 are biomarkers associated with vitiligo. CD4 T Cell, CD8 T Cell, Tregs, NK cells, dendritic cells, and macrophages are related to vitiligo occurrence. These immune cells may hold a vital role in vitiligo development. Further exploration of the interaction between immune cell infiltration would help determine the immunotherapy goal for vitiligo and improve immunomodulatory therapy for vitiligo patients.

## Highlights

1. TYR, TYRP1, DCT, and LARP7 were selected as biomarkers associated with vitiligo.

2. Four biomarkers were associated with multiple immune cell infiltration in vitiligo.

3. TYR, TYRP1, DCT, and LARP7 were involved in the pathogenesis of vitiligo.
